# Glycerine in the House

**Published:** 1892-08

**Authors:** 


### GLYCERINE IN THE HOUSE.

This useful substance is almost exclusively used externally by housewives. It moistens and softens the skin, and, when properly diluted, both prevents and cures the painful and unsightly cracks known as "chaps" on the hands. We have seen it allay the excessive thirst of a fever when nothing else could do so. Two or three drops given to baby will often stop its stomach-ache, if wind be the cause. It will often soothe an irritable cough by moistening the dryness of the throat which gives rise to it. It is the most efficient means at our command for the prevention of bed-sores. M. Cattillon found by experiments that internally it increased the appetite and promoted nutrition. It has been found to be excellent as an enema in treating constipation. But if such uses are made of it, it must be pure and wholly unadulterated. Another use may be added, which is not generally known. When you are about to seal fruit-jars, drop in half a dozen drops of glycerine, and it will help in the keeping, and prevent mould from gathering on the top. If you want to show your husband a little attention, place a bottle at his hand of equal parts of glycerine and bay rum, for use after his morning shave, and he will rise up and bless you. This, I know, for I have tried it and can recommend it. Glycerine is also excellent for rubbing into shoes as a preventive of wet feet, as well as to soften the leather and keep it in good condition.—*Housewife.*

